# Increasing the Acceptability of Insect‐Based Foods as Future Foods: A Comprehensive Review of Barriers, Strategies, and Pathways to Mainstream Adoption

**DOI:** 10.1111/1541-4337.70534

**Published:** 2026-06-18

**Authors:** Jose Miguel Alvarez Suarez, Andrea M. Liceaga

**Affiliations:** ^1^ Laboratorio de Investigación en Ingeniería en Alimentos (LabInAli), Departamento de Ingeniería en Alimentos, Colegio de Ciencias e Ingenierías Universidad San Francisco de Quito USFQ Quito Ecuador; ^2^ Laboratorio de Bioexploración, Colegio de Ciencias Biológicas y Ambientales Universidad San Francisco de Quito USFQ Quito Ecuador; ^3^ Protein Chemistry and Bioactive Peptides Laboratory, Department of Food Science Purdue University West Lafayette Indiana USA

## Abstract

Edible insects are increasingly recognized for their high nutritional value and favorable environmental profile, yet their acceptance (defined as the continuum from willingness to try and purchase to repeated consumption) in Western and globalized food systems remains limited by cultural, sensory, regulatory, and economic barriers. This narrative review critically synthesizes recent advances in understanding the multidimensional determinants of consumer acceptance of insect‐based foods and evaluates the technological, cultural, and policy‐driven strategies proposed to overcome them, drawing on interdisciplinary evidence from food science, consumer behavior, and sustainability research.

Evidence indicates that processing approaches such as protein hydrolysis, extrusion, fermentation, and encapsulation can significantly improve sensory quality, functionality, and product integration into familiar food matrices. In parallel, targeted marketing, consumer education, and transparent regulatory frameworks emerge as essential to building trust and reducing neophobia. This narrative synthesis provides a comprehensive conceptual framework for advancing the mainstream adoption of insect‐based foods. The analysis highlights research gaps related to sensory optimization, traceability, sustainability assessment, and cross‐cultural consumer studies, offering evidence‐based directions to support the development of safe, acceptable, and nutritionally valuable insect‐derived products within contemporary food systems.

## 1 Introduction

Edible insects have long formed part of the human diet, particularly in Africa, Asia, and Latin America, where entomophagy remains intertwined with cultural identity, traditional knowledge, and ecological adaptation. While a growing body of literature has addressed specific dimensions of edible insect adoption—such as consumer attitudes and acceptance drivers (Olivadese and Dindo 2023), technological processing strategies and techno‐functional optimization (Liceaga 2021, 2019), or regulatory and safety frameworks (Alejandro Ruiz et al. 2025; Baiano 2020)—these studies largely remain fragmented and domain‐specific. Recent reviews have typically focused on isolated aspects, without fully integrating cultural, technological, communicative, and governance perspectives into a unified analytical framework. What remains missing is a holistic, interdisciplinary synthesis that connects these dimensions to explain not only why barriers persist, but also how coordinated technological, regulatory, and sociocultural strategies can jointly accelerate normalization and market integration. In these entomophagy regions, insects are consumed both as everyday foods and as seasonal delicacies, celebrated for their taste, nutritional richness, and role in sustainable livelihoods. By contrast, in most Western societies, insects have historically been excluded from mainstream diets and instead associated with disgust, poverty, or exotic practices (Olivadese and Dindo 2023). This duality underscores the paradox of insects as both a heritage food and a modern frontier of food innovation. At the same time, several studies indicate that urbanization and increasing exposure to Westernized lifestyles can reshape traditional entomophagy practices, although the magnitude and direction of these changes vary markedly across sociocultural contexts, signaling that acceptance challenges are not confined to Western societies (van Huis et al. 2013). While previous reviews have primarily examined these dynamics through isolated lenses—focusing separately on consumer attitudes, technological feasibility, or sustainability narratives—the present review advances beyond these fragmented approaches by integrating cultural, technological, communicative, and governance dimensions into a unified analytical framework that generates actionable insights for accelerating normalization and market integration.

Renewed scientific and commercial attention to edible insects is driven by their potential to strengthen food and nutrition security in the face of climate change, biodiversity loss, and rising global protein demand. Insects are widely recognized as a rich source of high‐quality proteins with generally balanced amino acid profiles. Nevertheless, their nutritional quality shows marked variability across species, developmental stages, rearing substrates, and processing technologies, which collectively influence protein content, amino acid composition, and digestibility. In particular, the presence of chitin and its interactions within the food matrix may impose constraints on protein bioaccessibility and bioavailability, highlighting the need for optimized processing strategies to enhance nutritional performance (Liceaga 2022, 2021; Alejandro Ruiz et al. 2025). Their production systems often exhibit high feed conversion efficiency, low direct water requirements, and reduced greenhouse gas emissions relative to conventional livestock; however, these advantages are highly sensitive to life cycle assessment assumptions and system boundaries (Halloran et al. 2016), including factors such as energy requirements for temperature control, feed composition, and processing conditions, all of which can substantially influence overall environmental performance. Together, these nutritional and environmental advantages position insects as one of the most promising alternative protein sources for advancing sustainable and resilient diets (Gebreyes and Teka 2025).

Yet, the promise of insects as sustainable proteins stands in stark contrast to their slow integration into Western and globalized diets. Despite advances in insect farming and food technology, a persistent adoption barrier hampers large‐scale diffusion. Consumer research consistently highlights ambivalence. For example, many consumers express curiosity about the sustainability and nutritional potential of insect‐based products, yet actual purchase intention and repeated consumption remain limited (Roccatello et al. 2024). Such hesitation is shaped by food neophobia, cultural perceptions of disgust, and negative sensory expectations, particularly when insects are offered in visible/recognizable, intact form (Acheampong et al. 2025). This discrepancy between technological feasibility and consumer acceptance has important implications. Although insect‐based products are increasingly entering international markets in the form of protein powders, snacks, baked goods, and beverages, their market incursion remains limited—particularly in Western markets—compared with other alternative proteins such as plant‐based analogues or cultured meat (Lin et al. 2025). This gap highlights that technological progress alone is insufficient to achieve widespread adoption.

In this context, the present review synthesizes recent advances in understanding and enhancing the acceptability of insect‐based foods. We examine psychological, cultural, and sensory barriers; technological and formulation strategies; marketing and communication approaches; and the role of regulatory frameworks in fostering consumer trust. By integrating insights from food science, consumer science, and policy studies, this review seeks to map the multidimensional determinants of insect food acceptance and to highlight promising pathways for future research and innovation. Building on this background, the objective of this review is to provide a roadmap for transforming edible insects from a niche novelty into a widely accepted element for a sustainable diet, capable of reconciling cultural heritage with future food innovations and inspiring more inclusive and resilient food systems. Beyond synthesizing existing evidence, this review also outlines actionable strategies to move edible insects from marginal acceptance toward mainstream adoption.

## 2 Methodological Approach

This review adopts a narrative and integrative methodological framework aimed at synthesizing multidisciplinary evidence on the acceptability of insect‐based foods, spanning food science, consumer behavior, sensory science, marketing, regulatory studies, and sustainability research. A literature search was conducted in the Web of Science, Scopus, and Google Scholar databases to identify studies addressing edible insects and consumer acceptance. The literature identification process was carried out iteratively during the manuscript preparation period, with database searches conducted between April and August 2025 across the three platforms to ensure coverage of the most recent literature. The search covered publications dating from January 2000 to August 2025, with particular emphasis on studies published after 2015, reflecting the rapid expansion of research on alternative proteins, edible insects, and consumer acceptance. The search strategy combined keywords related to edible insects, consumer behavior, and food innovation. The main search terms included “edible insects”, “entomophagy”, “insect‐based foods”, “consumer acceptance”, “food neophobia”, “consumer perception”, “insect protein”, “insect‐based products”, “sustainability”, and “alternative proteins”, which were combined using Boolean operators (AND, OR). For example, the following Boolean query was used in Web of Science and Scopus: (“edible insects” OR entomophagy OR “insect‐based foods” OR “insect protein”) AND (“consumer acceptance” OR “consumer perception” OR “food neophobia”) AND (sustainability OR marketing OR regulation OR “processing technologies”). Searches were conducted in the title, abstract, and keywords fields when possible. Both experimental and review studies addressing consumer perception, technological processing, regulatory aspects, and market development of edible insect products were considered. Additional relevant studies were identified through manual screening of reference lists from key publications. For Google Scholar, results were sorted by relevance, and the first 200 results (approximately the first 20 pages) were screened, following commonly recommended practices for improving reproducibility in narrative reviews. Studies not written in English, conference abstracts without full text, and publications not directly related to food applications of insects were excluded. Although non‐peer‐reviewed sources were generally excluded, authoritative reports from recognized international and regulatory organizations were considered when relevant, as they provide essential policy and safety frameworks for edible insects. These included reports and technical documents from organizations such as the Food and Agriculture Organization (FAO), World Health Organization (WHO), European Food Safety Authority (EFSA), Codex Alimentarius Commission, and food safety agencies. Such documents were treated as grey literature exceptions due to their institutional authority and relevance to regulatory and policy contexts.

Inclusion criteria comprised peer‐reviewed original research articles, systematic and narrative reviews, meta‐analyses, and authoritative reports from recognized international and regulatory organizations (e.g., FAO, WHO, EFSA, Codex Alimentarius, and national food safety agencies) published in English that addressed at least one dimension of insect‐based food acceptability, including psychological, cultural, sensory, technological, regulatory, economic, ethical, or environmental aspects. Exclusion criteria included purely descriptive entomological studies lacking food‐system relevance, non‐peer‐reviewed sources, conference abstracts, and market reports without methodological transparency. Retrieved publications were first screened based on title and abstract for relevance, followed by full‐text evaluation. Eligible studies were then qualitatively synthesized and categorized into major analytical domains, namely: (i) cultural and psychological barriers, (ii) sensory drivers and technological mitigation strategies, (iii) regulatory and safety frameworks, (iv) economic and market constraints, (v) ethical and environmental considerations, and (vi) marketing, communication, and education approaches. This thematic structuring enabled the identification of cross‐cutting interactions among barriers and strategies, supporting the development of an integrated conceptual framework for understanding the multidimensional determinants of consumer acceptance.

Rather than aiming for exhaustive quantitative synthesis, this narrative approach prioritizes conceptual integration, critical interpretation, and translational relevance, allowing the formulation of actionable pathways for innovation, policy development, and market adoption. This framework is particularly suited to complex, interdisciplinary topics—such as edible insect acceptance—where consumer behavior, food technology, governance, and cultural dynamics intersect and co‐evolve.

## 3 Barriers to the Acceptance of Insect‐Based Foods

**FIGURE 1:**
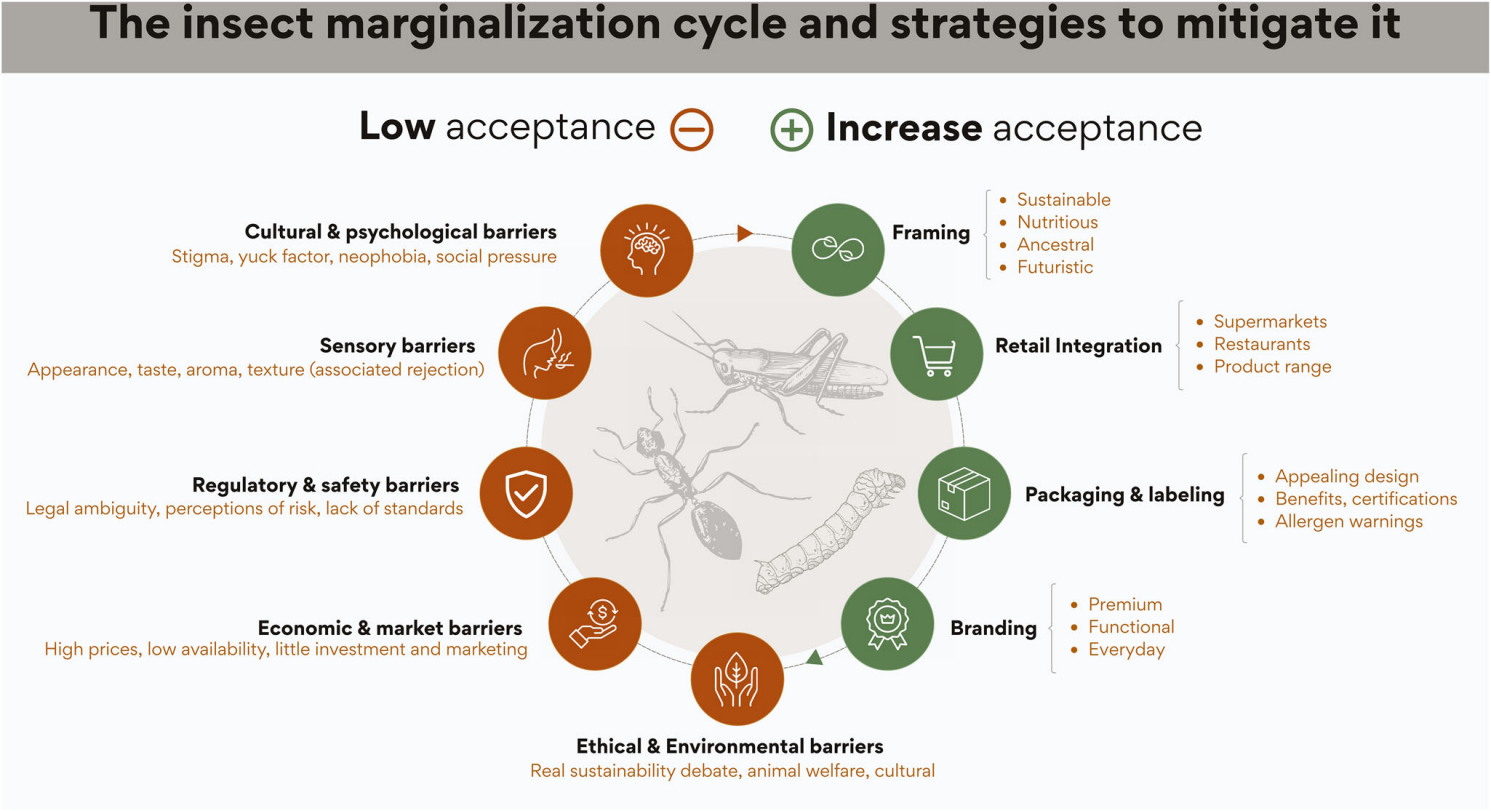
The insect marginalization cycle (interactions among cultural, sensory, regulatory, ethical, and economic barriers create a self‐reinforcing cycle of low consumer acceptance of insect‐based foods) and marketing, communication, and consumer education strategies for insect‐based foods.

Despite a growing body of evidence on their nutritional and environmental advantages, edible insects continue to face persistent obstacles that hinder their widespread acceptance in modern food systems. These barriers are not isolated; rather, they interact dynamically, reinforcing one another in ways that create self‐perpetuating cycles of marginalization. For instance, cultural stigma amplifies sensory rejection, regulatory ambiguity fuels perceptions of risk, economic constraints restrict availability, and ethical concerns add further layers of skepticism. Together, they sustain unfamiliarity, neophobic attitudes, and limited demand as conceptualized in the integrative framework (**Figure** **1**), which synthesizes multidisciplinary empirical evidence by mapping recurrent barriers and their dynamic interdependencies into a unified model. Understanding these interdependencies is essential to developing strategies that can shift insects from being niche or novelty products into recognized and trusted protein sources. The following subsections (cultural and psychological, sensory, regulatory and safety, economic and market, and ethical and environmental) discuss these barriers in detail before considering how their interactions combine to perpetuate marginalization. This structure provides the foundation for exploring technological and policy innovations aimed at disrupting the barrier cycle and enhancing consumer acceptance.

### 3.1 Cultural and Psychological Barriers

Culture plays a central role in shaping food choices. In societies with long‐standing entomophagy traditions, such as regions of Africa, Asia, and Latin America, insects remain embedded in culinary heritage and social identity, often regarded as delicacies and seasonal foods (Olivadese and Dindo 2023). However, these practices exhibit substantial within‐region heterogeneity, shaped by urbanization, socioeconomic conditions, ethnic diversity, and the growing influence of modernization and Westernized dietary patterns. Ethnographic and nutritional studies across Mexico, the Amazon basin, and Central Africa consistently highlight that insects are not perceived as emergency foods, but rather as culturally valued and nutritionally relevant components of local diets (Chimbo‐Gándara et al. 2024; Cohen et al. 2009; Granados‐Echegoyen et al. 2024; Jaramillo‐Vivanco et al. 2024).

Conversely, in Western contexts with no historical precedent of entomophagy, insects are frequently associated with contamination, pests, and disease vectors, eliciting strong disgust reactions and negative affective responses (Kröger et al. 2022; Olivadese and Dindo 2023). This reaction, conceptualized as the “yuck factor,” constitutes one of the most powerful psychological barriers to acceptance. Disgust sensitivity, food neophobia, and social norms strongly modulate willingness to try insect‐based foods, with higher resistance typically observed among older, less educated, and risk‐averse consumers; while younger, environmentally conscious, and novelty‐seeking individuals display greater “willingness to try” insects (Roccatello et al. 2024; Szlachciuk and Żakowska‐Biemans 2024). Furthermore, repeated exposure, social modeling, and positive framing can significantly attenuate disgust responses and enhance acceptance, underscoring the dynamic and malleable nature of these psychological barriers.

Recent studies conducted in European frameworks confirm these trends. For example, survey data from 1017 Polish adults showed that neophobia was significantly higher among older and less educated individuals, who also tended to prefer “safe” and bland foods while paying less attention to food labelling information, particularly regarding price and shelf‐life (Jezewska‐Zychowicz et al. 2021). Furthermore, a nationally representative survey of 1,000 Polish consumers demonstrated that highly neophobic respondents perceived insect‐based foods as disgusting and risky, whereas those with lower neophobia described insect proteins as innovative, natural, and environmentally friendly. These respondents also expressed greater willingness to consume insect‐based ingredients when incorporated into processed forms within familiar food matrices, such as breads, snacks, or protein bars, rather than when presented as visible/recognizable insects (Szlachciuk and Żakowska‐Biemans 2024).

Beyond individual psychology, social norms exert a strong influence on consumer behavior. Consumers are more willing to try insect‐based foods when tasting occurs in social settings where the behavior is normalized. Moreover, peer and expert influences also significantly increase acceptance when endorsed by groups such as chefs/culinary opinion leaders, nutrition professionals, and food scientists. Such endorsements are particularly effective among individuals with lower disgust sensitivity, as these groups are perceived by consumers as credible sources of sensory, health‐related, and safety‐related information (Berger et al. 2019; Roccatello et al. 2024). Recent evidence further confirms that social signaling and normative cues strongly shape willingness to consume, underscoring that attitude toward insects is not solely a matter of individual taste but also of identity building and cultural framing.

### 3.2 Sensory Barriers

Sensory attributes are among the strongest predictors of consumer rejection or acceptance. While nutritional and environmental arguments may raise curiosity, actual consumption decisions are primarily driven by appearance, aroma, flavor, and texture. Importantly, recent comparative reviews integrating consumer surveys, controlled sensory panel evaluations, and market studies indicate that perceived inferior sensory quality relative to conventional meat remains the leading product‐related barrier for alternative proteins such as insects and cultured meat, particularly in minimally processed and visible product formats (Lin et al. 2025). This underscores that, without achieving parity in sensory appeal, especially in terms of flavor and texture, large‐scale adoption will remain elusive.

#### 3.2.1 Visual Appearance

**FIGURE 2:**
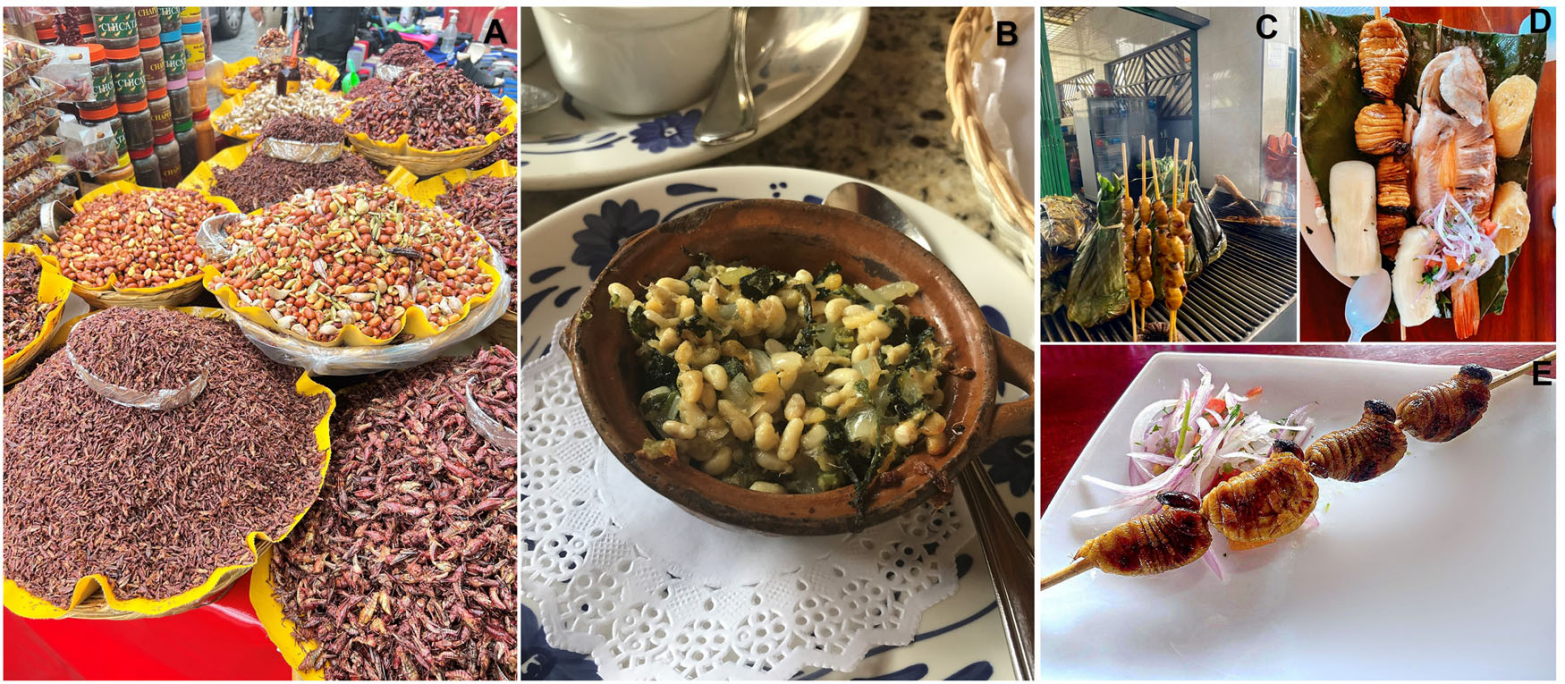
Traditional and contemporary culinary expressions of edible insects across cultures. Variety of chapulines at a market in Oaxaca, Mexico; (B) Escamoles in Mexico City; (C) heat‐roasted chontacuro (*R. palmarum*) larvae prepared for direct consumption in a popular market in the Ecuadorian Amazon. (D) *Maito*, a traditional Amazonian dish in which bijao (*kwan panga*) leaves are wrapped and tied with vegetable fiber, then cooked over fire with ingredients such as fish, hearts of palm, chontacuro, boiled green plantain, and cassava. (E) Gourmet preparation of heat‐roasted chontacuro larvae served with a traditional Ecuadorian pickle made of chopped onion, tomato, cilantro, and lemon juice (photos by the authors).

Appearance often acts as the first filter for acceptance of food. Insects such as crickets, grasshoppers, or mealworms trigger immediate aversion among consumers unfamiliar with entomophagy, primarily due to their recognizable morphology and associated disgust responses (Alhujaili et al. 2023). Even when processed, insect flours can impart darker coloration, altered texture, and distinctive flavor and aroma profiles to baked goods or pasta, which in Western sensory frameworks may evoke associations with burnt or spoiled foods (Bawa et al. 2020; Mierzejewska et al. 2025). By contrast, in societies with strong entomophagy traditions, these same sensory attributes are often perceived positively and linked to cultural identity and culinary authenticity. **Figure** **2** illustrates representative examples of traditional market displays and culinary presentations, highlighting how morphological visibility, presentation format, and visual exposure contribute to the normalization and positive framing of insect‐based foods. Consequently, acceptance pathways are highly context‐dependent. For instance, while product visibility and characteristic sensory cues reinforce authenticity in traditional settings, masking strategies such as fine milling, protein isolation, or incorporation into familiar food matrices are critical for enhancing consumer trust and sensory acceptance in novel markets, as consistently demonstrated by controlled choice and tasting studies comparing visible and nonvisible product formats (Yazici and Ozer 2021).

#### 3.2.2 Aroma and Flavor

Aroma and flavor are critical determinants of consumer acceptance of edible insects, as volatile compounds generated during processing and storage directly shape hedonic perception. Key drivers of rejection are typically associated with lipid oxidation products (e.g., aldehydes and nitrogen‐containing heterocycles) that can impart rancid, musty, grassy, or burnt notes, which are consistently rated negatively in sensory evaluations (Adámek et al. 2020; Liu et al. 2022; Ribeiro et al. 2024). Contrarywise, moderate thermal processing can generate Maillard‐derived compounds (e.g., alkylpyrazines) that can confer roasted, nutty, and savory aromas, which are more positively perceived and can partially mask undesirable flavors (Park et al. 2024; Reale et al. 2023; Żołnierczyk and Szumny 2021).

Species‐ and processing‐dependent differences in volatile profiles further modulate acceptability. For example, cricket, mealworm, and buffalo worm flours exhibit distinct aroma signatures that influence product formulation strategies, while excessive roasting temperatures intensify burnt and bitter notes, ultimately reducing sensory acceptance (Adámek et al. 2020; Reale et al. 2023). Collectively, these findings indicate that controlling lipid oxidation, optimizing thermal processing, and tailoring insect species selection are pivotal strategies for minimizing sensory drivers of rejection and enhancing consumer acceptance of insect‐based foods.

Applications in food matrices further highlight opportunities for sensory optimization. The incorporation of cricket powder into gluten‐free bread enhanced aroma complexity, producing caramel, roasted, and cooked potato notes attributed to pyrazines, which improved consumer acceptance (Wieczorek et al. 2022). However, insect proteins often retain a persistent earthy or “insect‐like” flavor that is not always successfully masked, limiting consumer appeal (Pan et al. 2022). To overcome this, incorporating insect flours into bakery products such as bread, cookies, and crackers has been proposed as a strategy to render the insect component less perceptible and more familiar (Kröger et al. 2022). Sensory research further shows that 93.2% of young European consumers (*n* = 118; aged 18–30) reported higher acceptance of insect‐based foods when their natural appearance was concealed, based on a cross‐sectional survey combined with controlled sensory tasting sessions of commercial products, including protein bars, granola, chocolate bars, spreads, and extruded snacks (Willeke et al. 2025). This finding underscores the importance of visual masking and formulation strategies toward enhancing acceptance among Gen Z and Millennial consumers.

#### 3.2.3 Texture and Mouthfeel

Texture and mouthfeel present another critical challenge. At the particle level, chitin‐rich exoskeleton residues act as insoluble inclusions that generate sandy or gritty sensations in baked goods or beverages, which are perceived as unpleasant in Western markets. However, at the matrix level, insect incorporation also modifies dough rheology and starch–protein interactions, leading to starch dilution, altered water binding, and lipid redistribution, which collectively reduce gas retention, limit expansion, and increase hardness. For instance, the inclusion of *T. molitor* flour in bread formulations decreased loaf volume and increased crumb hardness and perceived graininess, leading to lower sensory acceptability compared with control breads (Verni et al. 2025). Similarly, in extruded snacks, higher insect inclusion levels decreased expansion ratios and increased product hardness and bulk density, thereby compromising crispness and contributing to consumer rejection (Yuan et al. 2022). Further examples on the textural effects of adding edible insects to foods such as cereal products will be discussed in Section 4.1 and Table 4.

To address these challenges, several technological strategies are being explored. Enzymatic protein hydrolysis has emerged as one of the most promising approaches for improving solubility, foaming, and emulsifying properties, enhancing digestibility, and modulating allergenic potential in a species‐, enzyme‐, and process‐dependent manner. Functional properties of insect protein hydrolysates are highly dependent on experimental and processing conditions, including pH, ionic strength, defatting status, protein purity, enzyme specificity, and degree of hydrolysis, which limits direct quantitative comparisons across studies. For example, cricket protein hydrolysates obtained from defatted protein concentrates and subjected to controlled enzymatic hydrolysis under near‐neutral pH and low ionic strength conditions have been shown to exhibit solubility values approaching 90% and markedly improved foaming and emulsifying properties compared with unhydrolyzed proteins (Hall et al. 2018; Liceaga 2019). Recent studies have further unveiled the role of cricket protein fractionation in determining techno‐functionality. A comparative analysis of different body parts of *A. domesticus* revealed that protein concentrates from legs, thorax, and abdomen differ substantially in solubility, oil‐ and water‐holding capacity, and emulsifying ability, with thorax proteins showing the most promising profile for food formulations (Brena‐Melendez et al. 2025; Zielińska et al. 2018). Similarly, an evaluation of commercial processing methods showed that drying and milling conditions markedly affect the nutritional and functional properties of cricket powders and flours, altering their foaming, gelling, and dispersibility potential—factors directly linked to textural quality in baked and extruded applications (Brena‐Melendez et al. 2024). Other approaches include microencapsulation, which masks fibrous residues in beverages; extrusion optimization, where adjustments in temperature and moisture improve expansion and crispness; and blending with cereals or legumes, which enhances dough rheology and creates more familiar textures (Kröger et al. 2022).

Nevertheless, texture perceptions are not universal but rather culturally relative. For example, in Southeast Asia, crunchy textures are highly valued in snacks, and roasted crickets or bamboo worms are prized for their crispness (Gómez‐Corona and Valentin 2023). In Oaxaca, chapulines are enjoyed for their crunchy bite and smoky, umami‐rich flavor (Cohen et al. 2009), while in the Amazon, roasted palm weevil larvae (Chontacuro) (*R. palmarum*) are appreciated for their creamy mouthfeel and lipid richness (Jaramillo‐Vivanco et al. 2024). In Western Europe or North America, however, these same attributes often evoke disgust or perceptions of contamination (Tan et al. 2015).

This cultural asymmetry confirms that product development must integrate technological optimization—such as hydrolysis conditions, optimized processing, and blending strategies—to ensure acceptable textures in unfamiliar markets, while also leveraging the sensory attributes valued in entomophagous cultures as unique selling points for innovation and gastronomic diversity.

### 3.3 Safety and Regulatory Barriers

Beyond sensory and cultural dimensions, safety concerns and regulatory fragmentation represent structural barriers to consumer trust. As previously mentioned, many consumers, particularly in Western societies, associate insects with dirt, pests, or vectors of disease (Olivadese and Dindo 2023). These perceptions are partly cultural but also grounded in genuine biological and technological risks, which must be carefully addressed to ensure safe production and commercialization.

#### 3.3.1 Allergenicity and Cross‐Reactivity Risks

One of the most critical safety challenges relates to allergenicity, particularly the risk of cross‐reactivity with shellfish allergens. Several insect species contain homologous proteins such as tropomyosin and arginine kinase, which are well‐known allergens in crustaceans and mites. This is believed to be a result of the immunological relationships between crustaceans and arthropods (which include insects) (Liceaga 2022). For example, immunoinformatic data for shared sequence homology (>60% identity) of cricket tropomyosin and allergens from various species of shellfish, insects, and nematodes show top matches for tropomyosin from Pan b 1.0101 northern shrimp (*Pandalus borealis*) (Hall and Liceaga 2021). This molecular similarity can trigger IgE‐mediated allergic reactions in sensitized individuals, posing significant risks for consumers with existing shellfish allergies (Olivadese and Dindo 2023). Consequently, mandatory allergen labeling and rigorous risk assessment protocols are indispensable to protect vulnerable populations and to reinforce transparency and trust in insect‐based foods.

#### 3.3.2 Heavy Metal Bioaccumulation and Substrate Effects

Another major safety concern involves the bioaccumulation of heavy metals, including cadmium, lead, mercury, and arsenic, which can occur when insects are reared on contaminated substrates. The safety profile of insect products is therefore strongly shaped by the composition and quality of rearing substrates, as well as by species‐specific accumulation patterns. Poor substrate control and inadequate farming practices can lead to elevated contaminant levels, compromising food safety and regulatory compliance. These risks underscore the need for robust production standards, strict monitoring of feed materials, and validated traceability systems, particularly in informal or small‐scale production chains (Olivadese and Dindo 2023; FAO 2021).

#### 3.3.3 Microbial Hazards and Hygiene Risks

Microbial contamination represents an additional and highly relevant challenge. Insects may harbor pathogenic bacteria such as *Bacillus cereus*, *Pseudomonas aeruginosa*, *Staphylococcus aureus*, *Salmonella spp*., and *Escherichia coli*, as well as spore‐forming microorganisms that can survive processing if inadequate thermal treatments are applied (Liceaga 2019). Furthermore, inappropriate postharvest handling, storage, and processing conditions may facilitate microbial proliferation and mycotoxin formation. Critically, inadequate hygiene at artisanal or informal production stages exacerbates these risks, reinforcing consumer skepticism and rejection (FAO 2021). As such, the implementation of standardized hygiene protocols, validated processing technologies, and cold‐chain management is essential to ensure microbial safety and product stability.

#### 3.3.4 Regulatory Frameworks and Consumer Trust

Regulatory frameworks are not only technical instruments to guarantee food safety but also central drivers of consumer trust and acceptance. In the European Union, since 2015, the classification of insects as novel foods under Regulation (EU) 2015/2283 has provided a clear and harmonized pathway for commercialization (The European Commission 2015). To date, specific insect species and product forms—such as whole insects, frozen, dried, or powdered preparations, and in some cases partially defatted ingredients—have been authorized following case‐by‐case safety assessments. These include *Tenebrio molitor*, *Locusta migratoria*, *Acheta domesticus*, and *Alphitobius diaperinus*, together with mandatory allergen labeling, which signals to consumers that approved products meet the same rigorous standards as other food commodities (Turck et al. 2021, Turck et al. 2022, Turck et al. 2024, Turck et al. 2025). This regulatory transparency has been critical in legitimizing insect‐based products in European markets.

In North America, Canada recognizes certain insect species as non‐novel foods when there is evidence of traditional safe consumption, whereas the Food and Drug Administration (FDA) in the United States regulates insects under the Food, Drug, and Cosmetic Act. By subjecting edible insects to the same safety, labeling, and sanitary standards as conventional foods, the FDA positions insect products not as exotic or experimental, but as legitimate food items, a framing that directly influences consumer perceptions of normality and reliability. Similarly, across Asia, regulatory clarity also plays a role in consumer acceptance. In Thailand, edible insects are regulated under the Food Act B.E. 2522 (1979) and supervised by the Thai Food and Drug Administration, which establishes food safety standards, hygiene requirements, labeling rules, and market authorization procedures for both conventional and novel foods (Thai Food and Drug Administration 1979), while in South Korea they are governed by the Ministry of Food and Drug Safety (MFDS n.d.) and formally recognized as food ingredients under the Food Sanitation Act, reinforcing long‐standing entomophagy traditions and facilitating their transition into modern retail markets (Lähteenmäki‐Uutela et al. 2021). Moreover, in China, any new food raw material must undergo a formal safety evaluation and authorization by the National Health Commission (NHC) under the Administrative Measures for the Safety Review of New Food Raw Materials (NHFPC 2013). Within this regulatory framework, silkworm pupae (*Bombyx mori*) were formally approved in 2014, a milestone that has contributed to their normalization and incorporation into mainstream food products (Lähteenmäki‐Uutela et al. 2021). In Australia and New Zealand, edible insects fall under the Food Standards Australia New Zealand (FSANZ) Food Standards Code (FSANZ 2023), but the absence of insect‐specific provisions has prompted industry associations to issue voluntary guidelines, illustrating how self‐regulation attempts to fill regulatory gaps to maintain consumer confidence. By contrast, in several regions of Latin America and sub‐Saharan Africa, the regulatory governance of edible insects remains fragmented, heterogeneous, or largely absent, resulting in the predominance of informal production and marketing channels. In Mexico, despite the long‐standing cultural and gastronomic importance of edible insects, no harmonized national regulatory framework specifically governing insect‐based foods currently exists, and commercialization largely occurs through traditional markets, street vendors, and regional value chains, with substantial subnational variability in hygiene practices and oversight (Alvarez‐Suarez and Liceaga 2025). These regulatory gaps persist across much of Africa and Latin America, where the insect trade is predominantly informal, though notable exceptions and emerging pilot regulations exist at national or subnational levels. This regulatory fragmentation reinforces consumer uncertainty, constrains private‐sector investment, and hampers the transition of insect‐based foods from culturally embedded products into standardized, scalable, and mainstream food commodities (Alejandro Ruiz et al. 2025; Lähteenmäki‐Uutela et al. 2017; Lähteenmäki‐Uutela et al. 2021). Table 1 presents a structured comparative overview of these regional regulatory landscapes, which summarize regulatory status by region, approved species, responsible regulatory agencies, and key regulatory requirements, while also highlighting major regulatory gaps and persistent limitations.

**TABLE 1:** Global overview of regulatory status of edible insects.

Region/country	Regulatory framework type	Regulatory authority	Regulatory instrument(s)	Approved species/status	Key remarks
European Union	Harmonized authorization framework	European Commission; EFSA	Regulation (EU) 2015/2283 (Novel Foods Regulation)	Tenebrio molitor, Locusta migratoria, Acheta domesticus, Alphitobius diaperinus (specific product forms authorized)	Harmonized EU‐wide authorization system based on centralized EFSA safety assessment; species‐ and product‐form‐specific approvals; mandatory allergen labeling; high regulatory clarity and consumer trust
United States	General food law—no insect‐specific framework	FDA	Federal Food, Drug, and Cosmetic Act; FSMA; cGMP	No insect‐specific approvals; insects regulated as conventional foods	Insects subject to general food safety, adulteration, misbranding, and sanitation rules; absence of insect‐specific standards; regulatory gaps remain for species‐specific safety and allergen labeling
Canada	Case‐by‐case novel food authorization	Health Canada	Novel Foods Regulations (Food and Drug Regulations)	Species evaluated individually	Case‐by‐case authorization depending on demonstrated history of safe use or safety dossier; regulatory pathway similar to EU novel foods but without harmonized species lists
China	Case‐by‐case novel food authorization	National Health Commission (NHC)	Administrative Measures for the Safety Review of New Food Raw Materials (NHFPC 2013)	Bombyx mori pupae (approved in 2014)	Formal safety evaluation required for each new ingredient; silkworm pupae authorization facilitated integration into mainstream foods; other insects remain under evaluation
Thailand	National insect‐specific regulatory framework	Thai FDA	Food Act B.E. 2522 (1979); Ministerial Notifications	Multiple insect species	Long‐standing entomophagy integrated into formal food law; insect farming and processing governed by hygiene, safety, and labeling standards
South Korea	National insect‐specific regulatory framework	MFDS	Food Sanitation Act	Selected insect species recognized as food ingredients	Explicit inclusion of insects as food materials; strong institutional oversight supports market formalization
Australia and New Zealand	Case‐by‐case novel food authorization + voluntary self‐regulation	FSANZ	FSANZ Food Standards Code	Species assessed individually	Insects regulated under general food law; absence of insect‐specific standards; voluntary industry codes fill regulatory gaps
Mexico	General food law—no insect‐specific framework	COFEPRIS	General Health Law	No formal approvals	Long‐standing traditional consumption; commercialization mainly informal; absence of harmonized national standards; high subnational variability
Latin America (excluding Mexico)	General food law—no insect‐specific framework	National food safety agencies	National food laws	Highly heterogeneous	Predominantly informal markets; emerging pilot regulations in selected countries; fragmented governance
Sub‐Saharan Africa	General food law—no insect‐specific framework	National food authorities	National food safety laws	Highly heterogeneous	Traditional entomophagy widespread; regulatory oversight limited; commercialization largely informal; strong subnational variability

Abbreviations: CFIA, Canadian Food Inspection Agency; cGMP, current Good Manufacturing Practices; COFEPRIS, Federal Commission for the Protection against Sanitary Risk (Comisión Federal para la Protección contra Riesgos Sanitarios, Mexico); EFSA, European Food Safety Authority; EU, European Union; FAO, Food and Agriculture Organization of the United Nations; FDA, Food and Drug Administration (United States); FSANZ, Food Standards Australia New Zealand; FSMA, Food Safety Modernization Act (United States); GMP, Good Manufacturing Practices; HACCP, Hazard Analysis and Critical Control Points; MFDS, Ministry of Food and Drug Safety (Republic of Korea); NHC, National Health Commission (People's Republic of China); NHFPC, National Health and Family Planning Commission of the People's Republic of China (former name of NHC); WHO, World Health Organization.

#### 3.3.5 Regulation as a Driver of Acceptance and Market Normalization

Taken together, these examples illustrate that safety regulation is not only a prerequisite for risk management but also a powerful communication tool that actively shapes consumer acceptance. Clear regulatory approval signals legitimacy, reducing the perception of insect‐based foods as exotic or unsafe. Transparent allergen labeling not only informs but also builds trust by demonstrating that insect products are held to the same standards as conventional foods. Similarly, traceability systems—linking farming substrates, processing technology, and distribution chains—enhance credibility by showing accountability across the value chain.

Evidence from the European Union illustrates this dynamic, where the inclusion of insect species under the Novel Foods framework, together with standardized allergen warnings, has allowed companies to frame their products as safe, regulated, and trustworthy, directly influencing consumer willingness to try them. By contrast, in markets where regulations remain fragmented, uncertainty reinforces skepticism and discourages both investment and adoption. Thus, harmonization of global standards, robust hygiene protocols, and credible certification schemes are not merely safety imperatives; they represent critical pathways to normalize insect‐based foods, strengthen consumer confidence, and accelerate their transition from niche products to mainstream dietary options.

### 3.4 Economic and Market Barriers

From an economic perspective, cost remains one of the most critical barriers limiting the large‐scale adoption of insect‐based ingredients, particularly in comparison with established plant protein sources. Recent techno‐economic assessments and market surveys consistently indicate that insect powders are commercialized at prices one order of magnitude higher than conventional plant protein isolates. For example, in European and North American specialty markets, retail prices for cricket powder commonly range between ∼25 and 60 USD/kg, whereas bulk soy protein isolate typically falls within ∼2–6 USD/kg, corresponding to a 5–15‐fold price premium (Dossey et al. 2016; Lähteenmäki‐Uutela et al. 2021; Smetana et al. 2016). Similarly, in the EU market, mealworm (*Tenebrio molitor*) powders are commonly priced at ∼20–45 EUR/kg, compared with ∼3–7 EUR/kg for pea protein isolates, reinforcing the magnitude of current economic constraints for product formulation and competitiveness (Caparros Megido et al. 2016; Smetana et al. 2016).

A central limitation for consumer acceptance of insect‐based foods is therefore their weak price competitiveness relative to both plant‐based and conventional animal proteins. Production costs remain high due to limited scale, capital‐intensive infrastructure, energy requirements for climate control, and regulatory compliance, which collectively reinforce the perception of insect ingredients as premium or experimental products (Abro et al. 2025). By contrast, plant‐based proteins have benefited from rapid scaling, automation, and substantial private investment, which has driven continuous cost reductions and improved competitiveness across retail and food‐service sectors (Szenderák et al. 2022). Traditional meat, in turn, remains the price benchmark, supported by highly optimized production systems, mature logistics chains, and public support mechanisms (in some countries). Importantly, the structure, magnitude, and consumer‐price impact of agricultural subsidies vary substantially across regions and product categories, meaning that their influence on retail meat prices is highly context‐dependent (Graça et al. 2019; Leroy and Praet 2015). Market availability also reveals sharp asymmetries. Insect‐based products are largely confined to specialty shops, online platforms, and niche health‐food outlets, and their limited presence in mainstream retail channels sustains their perception as novelty items rather than everyday foods (Zaleskiewicz et al. 2024). In contrast, plant‐based analogues now enjoy widespread distribution across supermarkets, food‐service chains, and institutional catering, reinforcing their normalization as legitimate protein sources (Alcorta et al. 2021). Traditional meat remains universally accessible, with deep integration across retail outlets, restaurants, food service, and institutional procurement, which cements its role as the default protein choice in most societies (Possidónio et al. 2022).

Marketing has also played a decisive role in shaping consumer acceptance. For insects, branding strategies remain weak and market campaigns fragmented, which further reinforces perceptions of exoticism and unfamiliarity (Kröger et al. 2022; Puteri et al. 2023). This constrained visibility contrasts sharply with plant‐based companies, which invest heavily in advertising, strategic partnerships with fast‐food chains, and high‐profile branding campaigns, yielding substantially greater consumer awareness, trust, and trial rates (Szenderák et al. 2022). Importantly, marketing effectiveness in this sector is strongly driven by scale, brand coherence, and repeated consumer exposure, rather than mere promotional activity (Leroy and Praet 2015).

Institutional support further amplifies these differences. Insects remain marginal in schools, hospitals, or public procurement programs, keeping them niche and largely consumer‐driven (Puteri et al. 2023). Plant‐based foods, however, have gained visibility through integration into fast‐food chains and institutional menus, reinforcing their legitimacy and everyday presence (Venter de Villiers et al. 2024). Furthermore, traditional meat continues to enjoy longstanding institutional backing through government‐issued dietary guidelines, public procurement, and subsidies, which reinforce its legitimacy as an essential protein source (Graça et al. 2019). Finally, consumer perception mirrors these structural asymmetries. Insects are frequently seen as scarce, expensive, and culturally unfamiliar, which erodes trust and limits adoption (Olivadese and Dindo 2023). Meanwhile, plant‐based foods are increasingly perceived as accessible, affordable, and environmentally responsible, attributes that strongly align with consumer values and accelerate their mainstream acceptance (Szenderák et al. 2022). Traditional meat, despite concerns over sustainability and environmental impact, continues to be perceived as natural, tasty, nutritious, and culturally indispensable, reinforcing its position as the “gold standard” of protein consumption (Leroy and Praet 2015; Possidónio et al. 2022).

A comparative overview of these economic, market, and cultural dynamics is provided in Table 2, which summarizes how differences in price competitiveness, market availability, marketing strategies, institutional support, and consumer perception shape the acceptance of insect‐based, plant‐based, and traditional meat foods. When traditional meat is considered alongside plant‐based and insect‐based foods, the gap widens further, creating a dual disadvantage for insects. Unlike plant‐based foods, they lack affordability and scale, and unlike meat, they lack cultural familiarity and parity in sensory performance. Overcoming these asymmetries will require not only technological innovation and cost reduction, but also cultural repositioning and policy support to challenge the entrenched advantages of traditional meat.

**TABLE 2:** Comparative overview of economic, market, and cultural dynamics shaping consumer acceptance of insect‐based, plant‐based, and traditional meat foods.

Dimension	Insect‐based foods	Plant‐based alternatives	Traditional meat
Price competitiveness	Higher prices due to limited production, high investment costs, and lack of economies of scale → perceived as premium or experimental (Abro et al. 2025)	Declining costs thanks to scaling, automation, and investment → increasingly competitive with meat and dairy (Szenderák et al. 2022)	Generally affordable due to established large‐scale production, subsidies, and strong global infrastructure → remains price benchmark (Graça et al. 2019; Leroy and Praet 2015)
Market availability	Mostly in specialty shops, online platforms, or niche health‐food markets → low visibility in mainstream supermarkets (Zaleskiewicz et al. 2024)	Widely available in supermarkets, restaurants, and online → normalized as everyday options (Alcorta et al. 2021)	Universally available across retail, food service, and institutional channels → entrenched as default Protein (Possidónio et al. 2022)
Marketing strategies	Limited branding and fragmented campaigns → reinforce perception of novelty (Kröger et al. 2022; Puteri et al. 2023)	Aggressive marketing, fast‐food partnerships, and strong branding → framed as sustainable and aspirational (Szenderák et al. 2022)	Backed by powerful industry lobbies, strong cultural narratives, and consistent advertising → meat as tradition, and status (Leroy and Praet 2015)
Institutional support	Minimal integration into schools, hospitals, or procurement → remains niche (Puteri et al. 2023)	Broad adoption through partnerships with fast‐food chains and institutional menus (Venter de Villiers et al. 2024)	Longstanding integration in public procurement, subsidies, and dietary guidelines → reinforced as essential protein source (Graça et al. 2019)
Consumer perception	Seen as scarce, expensive, and culturally unfamiliar → low trust and adoption (Olivadese and Dindo 2023)	Seen as accessible, affordable, and environmentally responsible → increasing mainstream acceptance (Szenderák et al. 2022)	Perceived as natural, tasty, nutritious, and culturally indispensable, despite environmental footprint concerns (Leroy and Praet 2015; Possidónio et al. 2022)

### 3.5 Ethical and Environmental Barriers

Beyond cultural, sensory, and regulatory factors, ethical and environmental concerns represent emerging determinants of consumer acceptance. Although insects are often promoted as sustainable protein alternatives, skepticism remains regarding their true environmental benefits and the ethical implications of large‐scale insect biomass production. Life‐cycle assessments (LCAs) generally confirm that insect farming requires less land, water, and feed than conventional livestock, while also yielding lower greenhouse gas emissions and higher feed conversion efficiency, particularly when environmental impacts are normalized per kilogram of edible protein or per nutritional unit (e.g., per kilocalorie or per serving), rather than per unit of fresh mass (van Huis and Oonincx 2017). Positive potential is especially evident when insects are reared on by‐products or agro‐waste, yet concerns arise when production relies on unsustainable inputs, raising questions about the “real” sustainability of the sector (Smetana et al. 2016). However, outcomes vary widely depending on production scale, substrate choice, and processing methods, which complicates direct comparisons with plant‐based proteins and challenges simplistic sustainability narratives (Halloran et al. 2016). This variability fuels consumer doubt and creates space for accusations of “greenwashing,” which can undermine trust and slow market normalization.

At the same time, ethical issues are gaining increasing attention. While insects are phylogenetically distant from vertebrates and current neurobiological evidence suggests fundamentally different sensory and cognitive capacities, the extent to which insects experience pain, stress, or sentience remains actively debated rather than scientifically settled. Consequently, ethical discussions around insect welfare—encompassing questions of nociception, stress physiology, stocking density, handling practices, and slaughter methods—represent an evolving research frontier rather than an established regulatory or scientific consensus (Delvendahl et al. 2022; Mancini et al. 2019). For some consumer segments, particularly in high‐income markets where animal welfare considerations strongly shape dietary choices, this scientific uncertainty itself may contribute to ethical discomfort and hesitation, thereby influencing acceptance.

In parallel, Indigenous and local communities in Latin America, Africa, and Asia emphasize the biocultural value of insects, framing them not merely as nutritional resources but as integral components of ecosystems, cultural identity, traditional knowledge systems, and livelihoods (Omuse et al. 2024). Providing respect for these perspectives requires concrete mechanisms, including benefit‐sharing agreements, community‐based sourcing standards, participatory value‐chain governance, transparent provenance certification, and co‐branding strategies that visibly acknowledge Indigenous contributions. Failure to implement such mechanisms risks cultural appropriation, inequitable value capture, and erosion of trust, which could weaken consumer acceptance in both local and global markets. To better visualize these dual dimensions, Table 3 summarizes the main ethical and environmental barriers shaping consumer acceptance of insect‐based foods. Taken together, ethical and environmental considerations underscore that acceptance depends not only on product quality and safety but also on broader perceptions of legitimacy, responsibility, and social justice. Demonstrating genuine sustainability through transparent life‐cycle assessments, third‐party certifications, and circular bioeconomy practices can help counteract accusations of greenwashing. Likewise, embedding welfare‐oriented husbandry principles and formalized community engagement frameworks fosters ethical integrity and social credibility, thereby transforming potential sources of skepticism into drivers of trust and supporting the long‐term normalization of insect‐based foods.

**TABLE 3:** Ethical and environmental barriers influencing consumer acceptance of insect‐based food.

Dimension	Barriers	Implications for acceptance
Environmental footprint	LCAs show lower GHGs, land, and water use than livestock, but results vary by substrate and scale → uncertainty about “real” sustainability (van Huis and Oonincx 2017)	Perceptions of inconsistency or greenwashing may erode trust and slow mainstream adoption.
Resource circularity	Positive potential when insects valorize by‐products (e.g., agro‐waste), but concerns arise if fed with unsustainable inputs (Smetana et al. 2016)	Consumers prefer clear evidence that insects contribute to circular economy practices.
Animal welfare	Ethical debates around insect sentience, stocking density, and killing methods remain unresolved (Mancini et al. 2019)	Ethical discomfort can discourage uptake in high‐income, welfare‐conscious markets.
Biocultural integrity	Indigenous traditions valorize insects as cultural foods, but commercialization risks appropriation if not inclusive (Omuse et al. 2024).	Lack of cultural recognition may weaken legitimacy and social acceptance.

Abbreviations: GHG, greenhouse gases; LCA, life cycle assessment.

## 4 Technological and Formulation Strategies to Enhance Acceptability of Insect‐Based Foods

Food technology provides a powerful toolbox to overcome barriers to the acceptance of insect‐based foods. By modifying sensory attributes, masking undesirable characteristics, and embedding insects into familiar food formats, technological innovation can mitigate visual disgust, suppress off‐flavors, and improve textural quality. At the same time, processing enhances nutritional and functional properties, enabling the development of products that are both palatable and familiar. This section examines key strategies—including “invisibility” approaches, extrusion, fermentation, encapsulation, blending with familiar ingredients, emerging processing techniques, and gastronomic innovation—and evaluates their potential to transform consumer curiosity into repeated consumption.

### 4.1 The “Invisibility Strategy”: Flours and Protein Isolates

One of the most effective technological approaches to overcoming consumer resistance to insect‐based foods is the so‐called “invisibility strategy,” which consists of transforming whole insects into flours, protein concentrates, and hydrolysates that can be seamlessly incorporated into familiar food matrices, thereby minimizing visual, morphological, and textural cues associated with disgust and neophobia. From a processing perspective, this strategy relies primarily on fine grinding, controlled particle size reduction, color masking, and modulation of lipid content and oil absorption, which together play a central role in mitigating sensory rejection. Milling dried insects into fine flours produces powders with protein levels ranging from 45% to 70%, together with fibers, lipids, and minerals, enabling nutritional fortification while improving dispersion homogeneity and reducing coarse mouthfeel in cereal‐based matrices. Partial substitution of cereal flours with insect powders generally enhances protein density, amino acid balance, and micronutrient content, while maintaining acceptable sensory quality at moderate inclusion levels (5%–10%), as consistently reported in bread, biscuits, pasta, crackers, muffins, noodles, and extruded snacks (Ardoin et al. 2021; Azzollini et al. 2018; Bawa et al. 2020; Biró et al. 2019; Bresciani et al. 2022; Cabuk and Yilmaz 2020; Djouadi et al. 2022; Khatun et al. 2021; Khuenpet et al. 2020; Mafu et al. 2022; Osimani et al. 2018; Pauter et al. 2018; Piazza et al. 2023; Roncolini et al. 2019; Ruszkowska et al. 2022; Thongkaew et al. 2024; Zielińska et al. 2021). At higher incorporation levels (>15%–20%), however, darker color, increased hardness, reduced loaf volume, excessive cooking losses, and decreased expansion are frequently observed, underscoring the importance of optimized formulation and processing control to balance nutritional gains and sensory performance (Gantner et al. 2022; Xie et al. 2022). Similar technological trends have been documented across a wide range of insect species, including *Alphitobius diaperinus*, *Rhynchophorus phoenicis*, *Macrotermes spp*., and *Zophobas atratus*, demonstrating that particle size engineering and formulation strategies rather than insect species per se largely determine final product quality (Ayensu et al. 2019; Awobusuyi et al. 2020; Gaglio et al. 2021; García‐Segovia et al. 2020; Ogunlakin et al. 2018; Sriprablom et al. 2022; Ortolá et al. 2022). In parallel, protein isolation and enzymatic hydrolysis offer technologically versatile alternatives, as these processes significantly enhance emulsifying properties, water‐holding capacity, and sensory acceptability, while also generating bioactive peptides and contributing to reduced allergenicity, thereby improving both functional and nutritional performance in food matrices (Calzada Luna et al. 2021; Hall et al. 2018; Liceaga 2019, 2021). Collectively, these technological interventions illustrate how processing acts as an active tool for sensory mitigation rather than a mere formulation step, enabling insect flours, isolates, and hydrolysates to function as invisible fortifiers in conventional foods. As summarized in Table 4, moderate inclusion levels (5%–10%) consistently provide a practical and broadly applicable compromise between nutritional enhancement and sensory quality across multiple food matrices, although optimal inclusion levels ultimately depend on product category, formulation objectives, regulatory targets, and consumer sensory thresholds, supporting invisibility as a key entry strategy for mainstreaming insect‐based foods by lowering visual neophobia and facilitating cultural normalization (Tarahi et al. 2024).

**TABLE 4:** Applications of edible insects in cereal products: Nutritional, techno‐functional, and sensory effects.

Product	Insect	Inclusion level	Main results	Sensory/Technological limitations	Ref.
Bread	*Acheta domesticus*	5%–30%	↑ protein, amino acids, Fe, and P; improved nutritional profile	>15% ↓ volume, ↑ hardness, darker color	(Bawa et al. 2020; Bresciani et al. 2022; Mafu et al. 2022; Osimani et al. 2018)
	*Tenebrio molitor*	5%–20%	↑ protein, amino acids, microbiological stability; acceptable up to 10%	>15% ↑ hardness, ↓ volume	(Jankauskienė et al. 2024; Gantner et al. 2022; Khuenpet et al. 2020; Roncolini et al. 2019; Xie et al. 2022)
	*Alphitobius diaperinus*	5%–10%	↑ protein and nutritional value; ↑ antioxidant activity	↓ dough extensibility; crumb darkening	(Gaglio et al. 2021; García‐Segovia et al. 2020; Igual et al. 2021)
	*Hermetia illucens*	2%–4%	↑ essential amino acids in dough and bread	↓ leavening capacity	(Montevecchi et al. 2021)
	*Locusta migratoria*	1%–5%	↑ protein, fat, and fiber; good sensory acceptance	Textural changes >5%	(Althwab et al. 2021)
	*Schistocerca gregaria*	10%–20%	↑ protein up to 60%	↓ volume; acceptable at 10%	(Haber et al. 2019)
	*Nauphoeta cinerea*	10%–15%	Improved nutritional profile without negative technical effects	Minor sensory differences	(de Oliveira et al. 2017)
Biscuits/Cookies	*Tenebrio molitor*	5%–30%	↑ protein, fiber, antioxidants	↑ hardness, ↓ spread ratio; darkening	(Xie, Yuan, et al. 2022; Zielińska and Pankiewicz 2020)
	*Alphitobius diaperinus*	13%–25%	↑ protein (“high in protein”)	↓ consumer acceptability (color, texture)	(Ortolá et al. 2022)
	*Rhynchophorus phoenicis*	35%–70%	↑ protein, fat, minerals	↓ carbohydrates and fiber; variable acceptability	(Ayensu et al. 2019)
	*Melanoplus cinereus*	5%–10%	↑ protein, fiber, Zn; improved acceptability	Color changes	(Dewi et al. 2020)
	*Macrotermes nigeriensis*	5%–20%	↑ protein and fat; ↓ carbohydrates and energy	Acceptable up to 5%	(Ogunlakin et al. 2018)
	*Macrotermes bellicosus*	5%–15%	↑ protein; good acceptability at 5%	↓ expansion >10%	(Awobusuyi et al. 2020)
	*Sitotroga cerealella*	5%	↑ protein, fiber, minerals	↓ dough stability	(Mohsen et al. 2024)
	*Zophobas atratus*	10%–30%	↑ nutrients, antioxidants	↑ hardness, darkening	(Sriprablom et al. 2022)
Crackers	*Tenebrio molitor*	2%–20%	↑ minerals; acceptable up to 6%	↓ crispness and structural expansion >10%	(Djouadi et al. 2022)
	*Acheta domesticus*	5%–20%	↑ protein; acceptable up to 15%	↑ hardness, darker color	(Ardoin et al. 2021)
	*Macrotermes bellicosus*, *Syntermes*, *Brachytrupes*	5%–15%	↑ protein; better acceptability at 5%	↓ color acceptability >10%	(Ardoin et al. 2021)
Muffins	*Tenebrio molitor*	1%–8%	↑ protein, phenolics	Darker crumb color, increased firmness	(Su‐Young Hwang and Choi Soo Keun 2015)
	*Locusta migratoria*	15%	↑ protein and baking yield	↓ volume	(Çabuk 2021)
	*Gryllodes sigillatus*	2%–10%	↑ protein, ↓ carbohydrates; ↓ glycemic index	Darker color	(Zielińska et al. 2021)
	*Acheta domesticus*	2%–10%	↑ protein, ↓ carbohydrates; stable acceptability	—	(Pauter et al. 2018)
	*Gryllus bimaculatus*	10%–40%	↑ protein, phenolics, antioxidants	>20% ↓ volume and acceptability	(Kim et al. 2024)
Pasta	*Acheta domesticus*	5%–14%	↑ protein, fiber; acceptable up to 5%–10%	↑ cooking losses	(Bresciani et al. 2022; Ho et al. 2022)
	*Tenebrio molitor*	15%	↑ protein, fiber, ash	↑ cooking loss; ↓ firmness and structural integrity; lower acceptability	(Çabuk and Yılmaz 2020)
	*Locusta migratoria*	15%	↑ protein, fiber	↓ acceptability compared with legumes	(Çabuk and Yılmaz 2020)
	*Bombyx mori*	5%–10%	↑ protein, ↓ energy; optimal acceptability at 10%	↑ acidity, ↑ cooking losses, ↓ firmness	(Biró et al. 2019; Piazza et al. 2023)
**Noodles**	*Acheta domesticus*, *Gryllus bimaculatus*, *Holotrichea sp*., *Gryllotalpa orientalis*	2%–10%	↑ tensile strength	↓ brightness (L*)	(Thongkaew et al. 2024)

*Note*: ↑ denotes an increase; ↓ denotes a decrease.

### 4.2 Extrusion and Texturization

Extrusion technology has become a pivotal method for developing insect‐enriched snacks, cereals, protein bars, and hybrid meat analogues. As a high‐temperature, short‐time process, extrusion not only enables the incorporation of insect ingredients into familiar food matrices but also profoundly modifies their techno‐functional and sensory properties, thereby shaping consumer acceptance. Importantly, the effects of extrusion strongly depend on the processing regime, particularly moisture content, allowing a clear distinction between low‐moisture extrusion (LME) and high‐moisture extrusion (HME), which lead to markedly different structural, textural, and sensory outcomes.

LME is the most widely applied approach for cereal‐based snacks and breakfast products fortified with insect flours. In this context, insect incorporation consistently enhances protein content and amino acid balance at moderate inclusion levels (5%–10%), while higher concentrations (>12.5%–15%) compromise expansion, crispness, and sensory quality. Studies with *Acheta domesticus* demonstrate that maize‐ and corn‐based extrudates maintain desirable puffing, crispness, and overall acceptability at 7.5%–10% cricket addition, whereas higher levels generate denser structures, increased hardness, and earthy off‐flavors that lower consumer acceptance (Igual et al. 2020; L. Ribeiro et al. 2021). Similarly, corn snacks enriched with 2%–8% cricket powder remained acceptable up to 6%, beyond which darker color, reduced expansion, and diminished sensory appeal were observed (Ruszkowska et al. 2022). Comparable trends have been reported for *Tenebrio molitor*, where 5%–10% addition improves nutritional value without major losses in product quality, but levels ≥15% lead to decreased expansion, gritty textures, and consumer rejection (Azzollini et al. 2018). Likewise, extrusion of maize blended with *Spodoptera purpurascens* flour achieves optimal sensory acceptance at 8%–10%, while higher incorporation levels induce excessive hardness and flavor imbalances (Ramírez‐Rivera et al. 2021).

A key mechanistic factor underlying these effects is the presence of chitin, the major structural polysaccharide of insect exoskeletons. Chitin acts as an insoluble dietary fiber that disrupts starch gelatinization and melt viscoelasticity during extrusion, thereby limiting bubble growth, reducing expansion, and increasing product density and hardness. Elevated chitin levels hinder the formation of continuous starch–protein matrices, restrict gas cell stabilization, and promote the development of compact, rigid structures, which collectively explain the loss of crispness and increased hardness observed at high insect inclusion levels (Azzollini et al. 2018; Igual et al. 2020).

In contrast, HME has opened new opportunities for structured insect‐based foods, particularly in the development of meat analogues. Under high water content and intense thermo‐mechanical shear, insect proteins can be reorganized into anisotropic, fibrous structures that closely resemble the texture of muscle tissue. When blended with soy protein isolate, cricket powder yields extrudates with well‐defined fibrous alignment, with cricket inclusion up to 30% still enabling fiber formation, while tensile strength, chewiness, and structural coherence are strongly modulated by barrel temperature, screw speed, and moisture content (Kiiru et al. 2020). More recent evidence indicates that incorporation of approximately 10% cricket powder optimizes fibrousness and chewiness, whereas higher levels weaken mechanical integrity and generate cracks and discontinuities in the extrudate matrix (Wang et al. 2024). These findings underscore the importance of precise moisture and thermal control to balance protein denaturation, molecular alignment, and phase separation during HME processing.

Taken together, current evidence demonstrates that extrusion provides a versatile platform for mainstreaming insect‐based foods, with LME primarily suited for cereal snacks and expanded products, where inclusion levels must be carefully optimized to avoid chitin‐driven losses in expansion and crispness, while HME enables the fabrication of fibrous, meat‐like analogues, offering consumers a familiar textural entry point into insect‐derived proteins. The addition of insect protein hydrolysates may further enhance techno‐functional and sensory outcomes. To synthesize these findings, Table 5 summarizes extrusion regimes, processing parameters, inclusion levels, nutritional and techno‐functional effects, and sensory outcomes, highlighting extrusion as a transformative technology for balancing functionality, sensory quality, and consumer acceptance through familiar snack and meat‐analogue formats. Optimizing extrusion conditions and formulation strategies thus represents one of the most scalable pathways for integrating insect proteins into daily diets.

**TABLE 5:** Selected extrusion parameters for insect‐enriched foods and their impact on texture and consumer acceptance.

Insect species/Ingredient	Food matrix	Extrusion conditions	Main outcomes (nutritional/techno‐functional)	Sensory/consumer effects	References
*Acheta domesticus* (cricket flour, 5%–20%)	Maize‐based extruded snacks	Low‐moisture extrusion; 120–160°C; 12–18% inclusion	↑ Protein density and amino acid balance; ↓ expansion and ↑ hardness (instrumental TPA)	Acceptable ≤10%; rejection >15% (sensory panel: hardness perception, aftertaste)	(L. Ribeiro et al. 2021)
*Acheta domesticus* (7.5%–15%)	Corn snacks	Low‐moisture extrusion; particle size reduced	↑ Protein; low WSI; ↓ expansion >12.5% (instrumental TPA)	7.5% retains crispness; >12.5% reduces crispness (sensory evaluation)	(Igual et al. 2020)
*Acheta domesticus* + soy protein isolate (up to 30%)	Meat analogues	High‐moisture extrusion; 120–160°C; water flow 9–10 mL/min	Fibrous anisotropic structures (instrumental tensile/shear analysis); ↑ tenderness (sensory evaluation) at 30% CP; high temp ↑ torsional resistance	Acceptable fibrous texture (sensory evaluation)	(Kiiru et al. 2020; Wang et al. 2024)
*Acheta domesticus* (2%–8%)	Corn snacks	Low‐moisture extrusion; CP 2–8% + optional baking powder	↑ Solubility; ↓ water absorption; ↓ expansion with ↑ CP (instrumental TPA)	Acceptable ≤6%; >6% rejection due to darker color and reduced expansion (sensory evaluation)	(Ruszkowska et al. 2022)
*Acheta domesticus* (cricket powder, 10%–50%) + soy protein isolate	SPI‐based meat analogs	High‐moisture extrusion; 140°C	↑ Anisotropic structure formation at 10% CP; ↓ complex modulus (G*) with ↑ CP content; ↓ mechanical strength and coherence at 20%–50% CP (cracks, insoluble components); ↑ pyrazines and ethers	Desirable roasted, nutty, and baked flavor profile from Maillard‐derived volatiles (flavor analysis); structural integrity compromised at excessive CP	(Wang et al. 2024)
*Alphitobius diaperinus* (protein concentrate, 15%–50%) + soy protein concentrate	Soy‐based meat analogs	High‐moisture extrusion; twin‐screw; 150–170°C; >40% moisture; with 5%–10% soy fiber	↑ Fibrous meat‐like structure; hardness and protein composition (25%–30.8%) comparable to meat (instrumental cutting/tensile analysis); optimal at 40% insect + 60% soy at 170°C	Meat‐like biting texture; improved fibrous appearance with soy fiber addition (SEM‐confirmed; instrumental texture evaluation)	(Smetana et al. 2018)
*Tenebrio molitor* (mealworm flour, 10%–20%)	Wheat‐based extrudates	Low‐moisture extrusion; 10–20% inclusion; high barrel temp and screw speed	10%: good expansion (instrumental TPA), source of protein (per EU claims); 20%: poor expansion due to fat (instrumental TPA)	Acceptable at 10%; rejection at 20% (hardness, low expansion) (sensory evaluation)	(Azzollini et al. 2018)
*S. purpurascens* (grasshopper meal, 0%–40%)	Maize‐based extrudates	Low‐moisture extrusion; 120–180°C; feed moisture ∼19%–21%	↑ Protein (up to 22.5 g/100 g); ↑ color change, browning; temp ↑ aroma, texture, flavor	Best acceptability at ∼8%–10% inclusion and ∼167°C; >20% decreased liking	(Ramírez‐Rivera et al. 2021)

*Note*: ↑ denotes an increase; ↓ denotes a decrease.

Abbreviations: CP, cricket protein/cricket powder (depending on experimental context); TPA, texture profile analysis; WSI, water solubility index;.

### 4.3 Fermentation and Flavor Improvement

Fermentation represents another powerful strategy to enhance both sensory quality and functional value of insect‐based foods. Lactic acid bacteria (LAB) fermentations of cricket and mealworm flours effectively suppress grassy and earthy notes while promoting the formation of lactic, buttery, and fruity volatiles, thereby reshaping overall aroma perception (Vasilica et al. 2022). At a mechanistic level, fermentation can alter volatile profiles by reducing aldehydes and other compounds associated with green and rancid notes, while generating desirable esters, organic acids, and alcohols that contribute to more pleasant sensory attributes. Beyond flavor modulation, fermentation enhances protein digestibility primarily by modifying matrix structure, reducing antinutritional interactions, and increasing enzyme accessibility, while in some systems, limited chitin depolymerization or physical disruption of chitin–protein complexes may further contribute to amino acid bioavailability (Goksen et al. 2025). Enzymatic pretreatment combined with fermentation further promotes the generation of bioactive peptides with antioxidant and antihypertensive activities (Hall et al. 2018, 2020; Malm and Liceaga 2021; Mendoza‐Salazar et al. 2021), pointing to promising applications in functional foods. Importantly, fermentation may also influence protein structure and epitope exposure, with potential implications for allergenicity, an aspect that remains insufficiently explored and warrants further systematic investigation.

In parallel, fermentation provides robust safety benefits and contributes to extended shelf‐life. In *T. molitor* pastes, lactic fermentation significantly reduced pH, inhibited spoilage and pathogenic microorganisms, and prolonged refrigerated shelf‐life up to eight weeks, outperforming conventional preservatives (Borremans et al. 2020). Likewise, fermentation of *Protaetia brevitarsis* larvae with lactic acid bacteria and yeasts reshaped volatile profiles, generating fruity and sweet esters that correlated strongly with higher consumer liking, particularly in yeast‐fermented samples (Cha et al. 2024). These findings underscore fermentation as both a preservation strategy and a sensory enhancer capable of substantially reducing consumer rejection of insect‐based foods.

Importantly, embedding insects into culturally familiar fermented matrices—such as bread, yogurt, or tempeh‐like foods—reduces perceived novelty while aligning insect ingredients with well‐established health and tradition narratives. For instance, tempeh prepared using *T. molitor* flours fermented with *Rhizopus oligosporus* improved iron bioavailability, thereby positioning insect proteins within a familiar plant‐based fermented format that enhances consumer acceptability (Wilson et al. 2024). Fermentation thus operates at both technological and cultural levels, simultaneously improving palatability, safety, and product legitimacy. Beyond solid foods, the incorporation of edible insect proteins into fermented beverages such as kombucha or kefir could further align entomophagy with the rapidly expanding market of functional drinks. Although direct applications of insects in these matrices remain unexplored, the established capacity of such fermentations to generate organic acids, bioactive peptides, and postbiotic compounds suggests a promising and innovative research direction.

Concrete food applications further strengthen this evidence. Lacto‐fermentation of cricket flour with *Lactiplantibacillus plantarum* and *Lacticaseibacillus casei* significantly improved the volatile profile of wheat biscuits, reduced acrylamide formation during baking, and maintained high sensory acceptability (Bartkiene et al. 2023). Likewise, sourdough bread fortified with 20% cricket powder and produced using selected LAB strains (*L. plantarum* CR L1 and *L. curvatus* CR L13) exhibited enhanced protein content, improved acidification performance, and increased microbial stability during backslopping (Galli et al. 2020). Collectively, these findings demonstrate that fermentation simultaneously optimizes safety, nutritional value, sensory performance, and cultural integration of insect‐enriched foods.

Taken together, fermentation emerges as a versatile technological platform for advancing insect‐based foods from niche to mainstream markets by reshaping volatile profiles, enhancing digestibility, improving safety, and embedding insect ingredients within culturally familiar formats. Recent advances in starter culture selection, enzymatic synergy, allergenicity modulation, and postbiotic generation highlight fermentation not only as a flavor‐enhancing technology but also as a foundation for developing insect‐derived functional foods with tangible nutritional and health‐promoting value. Future research should explicitly address the mechanistic links between fermentation‐driven biochemical transformations, sensory perception, and allergenicity, thereby enabling the rational design of fermented insect‐based foods that maximize both consumer acceptance and physiological benefits.

## 5 Marketing, Communication, and Consumer Education Strategies

Even when technological advances succeed in reducing sensory barriers, edible insect products will only achieve broad consumer acceptance if they are effectively communicated, marketed, and embedded into culturally resonant narratives. Acceptance of novel foods is rarely driven by intrinsic nutritional or environmental attributes alone; instead, it is mediated by psychological variables such as food neophobia and disgust sensitivity, as well as by external factors including trust, social norms, and culturally constructed meanings (Onwezen et al. 2021). Evidence from consumer research on emerging food technologies indicates that framing strategies primarily influence trial intention and initial willingness to taste, whereas branding and packaging shape purchase intention, perceived safety, and trust. Education‐driven interventions, in turn, play a central role in long‐term acceptance, repeat consumption, and habit formation (Siegrist and Hartmann 2020). In this context, marketing, communication, and education operate not merely as promotional tools but as behavioral levers that modulate sequential stages of consumer adoption, from curiosity and first exposure to sustained dietary integration. These interconnected strategies—framing, branding, packaging and labeling, and education/market integration—can be explicitly linked to measurable consumer outcomes, including trial, purchase, and repeat consumption. Where direct empirical evidence remains limited, these relationships are framed as testable hypotheses, highlighting critical research gaps and priorities for advancing evidence‐based marketing and communication strategies for insect‐based foods.

### 5.1 Framing and Narrative Construction

The way insects are framed—whether as sustainable, nutritious, ancestral, or futuristic—strongly shapes consumer responses and purchase intentions. Quantitative consumer research consistently shows that sustainability framing significantly increases willingness‐to‐try and willingness‐to‐pay (WTP) for insect‐based foods, particularly among environmentally conscious segments. Large‐scale surveys indicate that younger, urban, and sustainability‐oriented consumers exhibit 20%–40% higher acceptance and up to 25% higher WTP for alternative proteins, including edible insects, when environmental benefits are explicitly emphasized (Onwezen et al. 2021). Demographic segmentation further reveals that Millennials and Generation Z represent the most receptive consumer groups, driven by strong associations between dietary choices, climate responsibility, and ethical consumption.

Nutritional framing also exerts a measurable impact on consumer behavior. Emphasizing the high‐quality protein, iron, and omega‐3 content of edible insects significantly increases purchase probability, particularly in sports nutrition, functional foods, and health‐oriented markets (Liceaga 2022; Schiemer et al. 2018). Quantitative choice experiments show that positioning insect ingredients as performance‐enhancing rather than exotic can increase purchase intention by 20%–35%, especially among physically active consumers and health‐oriented segments. These findings highlight that nutritional messaging is particularly effective when aligned with functional and performance‐related consumption motives.

Heritage framing, which links insect consumption to Indigenous knowledge systems and traditional cuisines, further enhances consumer trust and perceived authenticity. Cross‐cultural studies indicate that cultural contextualization reduces perceived novelty and lowers food neophobia, particularly among consumers with strong interest in gastronomy, sustainability, and cultural identity (Swiderska et al. 2022). Multidimensional narratives integrating sustainability, nutrition, sensory appeal, and cultural authenticity consistently outperform single‐attribute frames, leading to significantly higher acceptance and engagement. Some studies report that certain product formats or communication strategies can increase consumers’ stated willingness to try insect‐based foods by up to twofold in specific experimental contexts, particularly when insects are incorporated into processed products or when sustainability benefits are emphasized (Syartiwidya et al. 2025). However, estimates of consumer acceptance vary considerably depending on the measured outcome (e.g., willingness to try, purchase intention, or willingness to pay), study design, and cultural context, and therefore should be interpreted as context‐dependent rather than a generalizable effect across all stages of the acceptance continuum (Wassmann et al. 2021).

These findings indicate that framing strategies are not merely communication tools but powerful levers shaping market segmentation, consumer trust, and purchasing behavior. By strategically combining environmental, nutritional, and cultural narratives, marketers, policymakers, and educators can effectively reposition edible insects from stigmatized products to credible, desirable, and socially accepted food options, thereby accelerating their transition from novelty to mainstream consumption.

### 5.2 Branding, Segmentation, and Positioning

Branding strategies play a pivotal role in shaping whether insects are perceived as niche novelties, functional foods, or mainstream dietary options. Beyond technological advances that improve product quality, it is branding and positioning that ultimately define the symbolic meaning consumers attach to them, making this dimension a key lever for expanding consumer acceptance. At the high end, premium branding has been particularly successful in upscale gastronomy, with chefs such as René Redzepi of Noma incorporating live ants and cricket pastes into tasting menus, thereby reframing insects as innovative and luxurious ingredients rather than survival foods (Danilovich 2023; The Guardian 2015). Importantly, such fine‐dining applications represent niche pathways with limited direct market penetration. Their primary contribution lies in symbolic legitimation, prestige signaling, and media amplification, which can indirectly influence mainstream acceptance by reshaping cultural meanings, enhancing perceived status, and engaging early adopters and opinion leaders. However, these prestige effects do not automatically diffuse into mass markets, underscoring the need for complementary strategies—such as functional branding, price accessibility, and integration into everyday food formats—to democratize acceptance and achieve large‐scale adoption. In this context, functional branding leverages the nutritional profile of insects to target athletes and health‐conscious consumers. Companies such as Entomo Farms in Canada and Exo in the USA promote cricket protein products—ranging from snack bars to protein powders—as high‐protein, sustainable alternatives aligned with sports nutrition and functional food trends, a positioning broadly consistent with industry‐based market forecasts suggesting potential growth of the global cricket protein powder sector from approximately USD 130 million in 2025 to USD 275 million by 2032; however, this estimate is included here only as contextual grey literature to illustrate commercial interest and investment dynamics, and should not be interpreted as part of the scientific evidence base given the limited methodological transparency of such reports (Persistence Market Research 2025). This example illustrates how insects can be framed not as exotic curiosities but as efficient, science‐backed solutions for modern dietary needs, aligning them with the rapidly growing market for sports and functional foods. At the everyday level, staple branding seeks to normalize insect consumption by embedding cricket or mealworm flours into bakery products, pasta, or extruded snacks. Yet this approach faces the challenge of meeting strong sensory expectations, since consumers demand taste, texture, and appearance comparable to conventional staples. Evidence shows that insect flours and powders are already incorporated into breads, cookies, noodles, and snacks to boost protein while masking insect visibility, but sustained acceptance depends on achieving parity with traditional sensory benchmarks (Cong et al. 2024; Onwezen et al. 2021). Furthermore, refining this strategy offers perhaps the most important and direct pathway to mainstream adoption, since repeated exposure to insect‐based staples in familiar formats can gradually reframe them as “normal” foods rather than novelties. Finally, segmentation research consistently identifies three broad consumer groups. Innovators and early adopters—typically young, urban, sustainability‐driven, and open to experimentation—influence broader social acceptance and act as opinion leaders in emerging food trends (Brunner and Nuttavuthisit 2019; Martins et al. 2022). Performance‐oriented consumers, such as athletes or protein‐conscious individuals, are motivated by functional benefits and nutritional efficiency (Martins et al. 2022). In contrast, resistant groups, including older, more conservative, or food‐neophobic consumers, tend to remain reluctant to adopt insect‐based foods, even when positively framed (Martins et al. 2022). Targeting early adopters through tailored branding can catalyze social contagion effects, triggering gradual expansion into mainstream markets (Brunner and Nuttavuthisit 2019; Martins et al. 2022). This progression—from aspirational fine dining to functional markets and eventually to everyday staples—suggests a phased branding strategy as a viable roadmap for moving insects from the margins of novelty toward broad consumer acceptance.

### 5.3 Packaging, Labeling, and Visual Communication

Packaging and labeling function as “silent marketers,” shaping consumer impressions before the product is tasted, and for insect‐based foods, this dimension is particularly sensitive, as it can either mitigate or amplify psychological barriers such as disgust. Empirical studies show that minimalist and clean‐label designs emphasizing protein content, naturalness, and sustainability outperform packages displaying explicit insect imagery, which often evoke negative associations among unaccustomed consumers (Naranjo‐Guevara et al. 2023; Onwezen et al. 2021). Neutral or abstract graphics and natural color palettes convey health and safety, while avoiding cues linked to pests or contamination, thereby facilitating first encounters with insect products. At the same time, positive communicative cues such as eco‐labels, nutritional claims (“high in protein,” “rich in iron and omega‐3”), and third‐party certifications (organic, fair trade, sustainable sourcing) have been shown to increase consumer trust and willingness to pay. Evidence from European markets indicates that willingness to pay rises significantly when nutritional claims are combined with certification logos, underscoring the role of third‐party validation in legitimizing insect‐based foods and embedding them within broader food quality standards (de‐Magistris et al. 2015).

Transparency also plays a central role, since allergen labeling—particularly concerning cross‐reactivity with shellfish—is indispensable both for regulatory compliance and for consumer protection. Importantly, effective risk communication requires a careful balance between clarity and reassurance. For instance, allergen warnings should be explicit, standardized, and prominently displayed, while complementary positive framing (e.g., sustainability, traceability, or quality claims) should be positioned separately to avoid diluting or obscuring safety information. Evidence from food risk communication research indicates that layered labeling strategies—combining mandatory, clearly visible allergen statements with optional, positively framed quality or sustainability cues—can enhance both perceived safety and trust without increasing anxiety or confusion. In this context, emerging digital tools such as QR codes linked to blockchain‐enabled traceability systems offer an additional layer of transparent information, allowing consumers to voluntarily access detailed sourcing, safety, and sustainability data while preserving label simplicity (Dobermann et al. 2017).

In this context, digital traceability should be viewed as a scalable continuum rather than a blockchain‐dependent solution. Cost‐effective and widely adopted alternatives—such as batch‐level traceability systems, QR codes linked to centralized databases, and third‐party certification and auditing schemes—offer practical, low‐barrier options particularly suited for small and medium‐sized enterprises (SMEs), enabling transparency, regulatory compliance, and consumer trust without high technological or financial burdens (Rossi et al. 2025). More advanced solutions, including blockchain‐enabled platforms, can provide additional layers of data immutability, interoperability, and real‐time integration, but their adoption should be considered context‐dependent rather than essential, depending on product complexity, supply‐chain scale, infrastructure, and governance requirements (Kamilaris et al. 2019).

Taken together, these findings indicate that packaging and labeling are not neutral carriers of information, but active levers in shaping consumer acceptance. Future strategies should therefore integrate standardized allergen disclosure, evidence‐based risk communication principles, and optional digital transparency tools, alongside minimalist and trust‐building design. Such approach aligns safety communication with consumer values of health, sustainability, and accountability, thereby facilitating the normalization of entomophagy and supporting the transition of insect‐based products from niche novelties into credible, everyday food options.

### 5.4 Consumer Education, Market Integration, and Ethical‐Technical Synergies

Education, retail integration, and ethical communication jointly constitute three interlinked mechanisms—exposure, familiarity, and framing—that underpin the long‐term normalization of edible insects. First, repeated exposure through education and experiential learning plays a central role in reducing disgust and food neophobia. School‐based programs, public workshops, tastings, and cooking classes consistently demonstrate that direct sensory engagement is more effective than passive information in reshaping attitudes, as positive tasting experiences and social interaction foster trust and willingness to consume insect‐based foods (House 2016; Jauniskis and Michopoulou 2021; Tan et al. 2015; van Thielen et al. 2019). This mechanism is further amplified when insects are introduced in collective and institutional contexts, such as universities or workplace catering, where peer influence and social normalization enhance trial and acceptance (Penedo et al. 2022). Second, cultural familiarity and embedding insects into known food systems emerge as powerful drivers of acceptance. Cross‐cultural evidence indicates that insects are readily accepted when incorporated into traditional dishes and familiar food matrices, thereby reducing perceived novelty and reinforcing identity, continuity, and sensory expectations. This mechanism is evident in regions where entomophagy is embedded in culinary heritage, as well as in Western contexts where insect ingredients are integrated into everyday staples such as bread, tortillas, snacks, or burgers, which significantly lowers psychological barriers and increases purchase intention (Puteri et al. 2023; Tao, and Li, et al. 2018; Halonen et al. 2022). These findings underscore that normalization is not achieved by exoticization, but by routinization within established dietary practices. Third, ethical framing and technological–communication synergies shape trust, legitimacy, and long‐term consumer engagement. Sustainability narratives require robust life‐cycle assessments to avoid greenwashing and maintain credibility (Dobermann et al. 2017), while respectful engagement with Indigenous food traditions is essential to prevent cultural appropriation and foster equitable value chains (Parodi et al. 2018; Swiderska et al. 2022). At the same time, alignment between technological innovation and communication strategies enhances resonance: fermented insect‐based foods can be framed as probiotic or gut‐friendly, protein hydrolysates positioned within nutraceutical markets, and extruded snacks marketed as sustainable alternatives to conventional products. Such synergies illustrate that acceptance is not solely a function of product quality, but of integrated sociotechnical design, where processing advances and narrative framing converge to meet consumer values and ethical expectations.

Taken together, exposure, familiarity, and ethical framing define a coherent roadmap for transforming edible insects from marginal novelties into credible, desirable, and socially legitimate food options. Future strategies should prioritize the co‐development of educational, technological, and communication interventions, thereby embedding insects within broader transitions toward sustainable, inclusive, and culturally grounded food systems.

## 6 Future Perspectives and Research Opportunities

**FIGURE 3:**
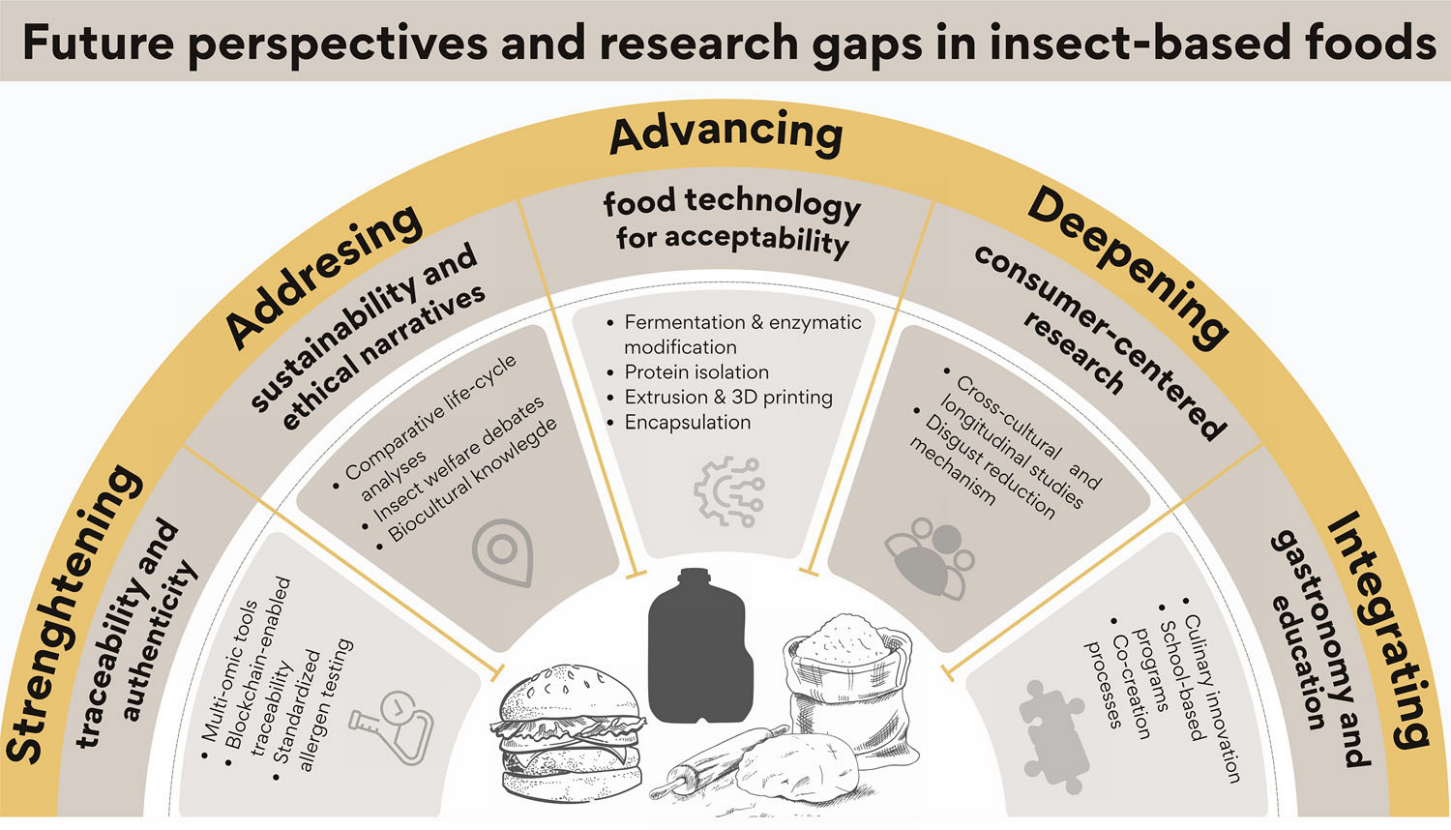
Main perspectives and research opportunities toward advancing the insect‐based foods.

Despite significant advances in nutritional characterization, processing technologies, and market positioning, the global acceptance of insect‐based foods remains constrained by persistent cultural, sensory, regulatory, economic, and ethical barriers. Moving forward, future research should shift from broad exploratory studies toward targeted, actionable priorities capable of accelerating large‐scale adoption and regulatory harmonization. To accomplish this, **Figure** **3** highlights five interrelated framework directions critical to advancing the insect‐based foods from niche products to globally accepted dietary components:

- Standardized sustainability assessment frameworks: Although insects are widely promoted as environmentally sustainable proteins, robust and comparable life‐cycle data remain scarce. Future work should prioritize the development of standardized life‐cycle assessment (LCA) and carbon footprint methodologies specifically adapted to insect farming systems, enabling consistent comparison across species, substrates, production scales, and geographic contexts. Such harmonized frameworks are essential to substantiate sustainability claims, guide technological optimization, inform regulatory decision‐making, and prevent accusations of greenwashing.
- Insect welfare guidelines and ethical governance: As production scales expand, ethical concerns regarding insect welfare are likely to gain prominence, particularly in high‐income markets. Research should therefore aim to establish science‐based welfare indicators, humane rearing and slaughter practices, and standardized ethical guidelines, analogous to those developed for livestock and aquaculture. Integrating welfare metrics into certification schemes and regulatory frameworks will be critical to maintaining consumer trust and ethical legitimacy.
- Optimized de‐chitinization and protein processing technologies: Chitin represents both a technological and nutritional challenge, influencing digestibility, allergenicity, texture, and sensory quality. Future technological innovation should focus on scalable, energy‐efficient, and food‐grade de‐chitinization strategies, coupled with optimized protein extraction and hydrolysis methods. These approaches should simultaneously target improved digestibility, reduced allergenicity, enhanced bioactivity, and cost competitiveness relative to plant protein isolates.
- Digital traceability and authenticity systems: Consumer trust and regulatory compliance increasingly depend on transparent traceability and authentication. Future research should integrate multi‐omic authentication tools (genomics, metabolomics, isotopic profiling) within tiered analytical frameworks, in which low‐cost screening methods are combined with advanced confirmatory techniques for high‐risk or disputed samples. Such hierarchical strategies are essential to balance analytical rigor with economic feasibility, particularly for small and medium‐sized enterprises (SMEs). These approaches should be coupled with digital traceability platforms—including QR codes, RFID, and blockchain‐enabled systems—to enable real‐time monitoring of species identity, geographic origin, substrate use, safety parameters, and sustainability metrics. However, blockchain‐based solutions should be regarded as complementary rather than essential, with adoption calibrated to product complexity, supply‐chain scale, infrastructure availability, and governance requirements. Moreover, while Codex Alimentarius and FAO technical discussions increasingly recognize the need for harmonized guidance on edible insects, globally unified regulatory standards remain under development, highlighting the importance of progressive, evidence‐based alignment rather than assumptions of imminent harmonization.
- Cross‐cultural and longitudinal acceptance studies: Current knowledge is dominated by short‐term, survey‐based studies conducted primarily in Western contexts. Future research should prioritize longitudinal, cross‐cultural, and intervention‐based studies to capture how repeated exposure, education, social norms, and generational change shape acceptance trajectories. Such approaches are essential to identify culturally adaptive strategies, reduce food neophobia, and design region‐specific market entry pathways. In parallel, the growing role of digital communication and social media warrants systematic evaluation, particularly regarding influencer‐driven dissemination, which can represent a practical mechanism for large‐scale engagement but also entails risks of misinformation, oversimplification, and sensationalism. Addressing these risks will require evidence‐based communication guidelines, expert co‐creation of content, and transparent disclosure standards.

Collectively, these five priorities define a focused and actionable roadmap for advancing insect‐based foods from niche products to globally accepted dietary components. Addressing these challenges in an integrated manner will be critical to ensuring that technological innovation, ethical governance, sustainability narratives, and consumer trust co‐evolve toward resilient, inclusive, and socially legitimate food systems.

## 7 Conclusions

Edible insects hold great promise as sustainable, nutritious, and culturally diverse protein sources, yet their mainstream acceptance remains constrained by interlinked cultural, sensory, regulatory, economic, and ethical barriers. These barriers interact to reinforce marginalization and delay market normalization, particularly in Western food systems. Advances in processing technologies, together with improved regulatory clarity, transparent safety standards, and inclusive communication strategies, create unprecedented opportunities to reposition insect‐based foods as safe, functional, and desirable products. Future research should prioritize three convergent directions: (i) consumer‐centered studies that integrate sensory science, behavioral psychology, and longitudinal exposure models; (ii) scalable and low‐impact processing technologies aligned with circular bioeconomy principles; and (iii) governance frameworks that harmonize food regulations, sustainability metrics, and equity‐oriented value chains. Aligning these efforts with global policy agendas—including the Sustainable Development Goals, IPBES nexus frameworks, and One Health strategies—will be essential to ensure that insect‐based foods contribute not only to sustainable protein transitions, but also to biodiversity conservation, public health, and social inclusion. Through coordinated innovation, governance, and market strategies, edible insects can evolve from niche products into credible components of resilient and future‐ready food systems.

## Author Contributions

**Jose Miguel Alvarez Suarez**: conceptualization, writing – original draft, writing – review and editing. **Andrea M. Liceaga**: conceptualization, writing – review and editing.

## Conflicts of Interest

The authors declare no conflicts of interest.
